# Hybrid Plasmonic/Photonic Nanoscale Strategy for Multilevel Anticounterfeit Labels

**DOI:** 10.1021/acsami.1c13701

**Published:** 2021-10-11

**Authors:** Vincenzo Caligiuri, Aniket Patra, Maria P. De Santo, Agostino Forestiero, Giuseppe Papuzzo, Dante M. Aceti, Giuseppe E. Lio, Riccardo Barberi, Antonio De Luca

**Affiliations:** † Department of Physics, University of Calabria, via P. Bucci, 31C, 87036 Rende, Cosenza, Italy; ‡ CNR Nanotec UOS Rende, via P. Bucci, 31D, 87036 Rende, Cosenza, Italy; § Istituto Italiano di Tecnologia, via Morego 30, 16163 Genova (GE), Italy; ∥ CNR-ICAR, Institute for High Performance and Networking, via P. Bucci 8-9c, 87036 Rende, Cosenza, Italy; ⊥ Institute of Electronics, Bulgarian Academy of Sciences, 72, Tsarigradsko Chaussee Blvd, 1784 Sofia, Bulgaria; # CNR-INO and European Laboratory for Non Linear Spectroscopy (LENS), Via Nello Carrara, 1, Sesto Fiorentino, 50019 Firenze (FI), Italy

**Keywords:** physical unclonable functions, Ag nanoislands, plasmonics, metal−insulator−metal structures, iridescence, structural colors, temperature exposure sensors

## Abstract

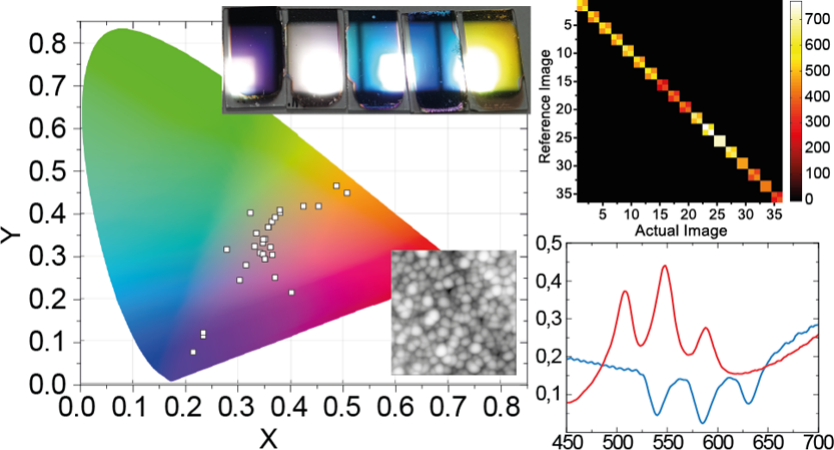

Innovative goods authentication strategies are of fundamental importance considering the increasing counterfeiting levels. Such a task has been effectively addressed with the so-called physical unclonable functions (PUFs), being physical properties of a system that characterize it univocally. PUFs are commonly implemented by exploiting naturally occurring non-idealities in clean-room fabrication processes. The broad availability of classic paradigm PUFs, however, makes them vulnerable. Here, we propose a hybrid plasmonic/photonic multilayered structure working as a three-level strong PUF. Our approach leverages on the combination of a functional nanostructured surface, a resonant response, and a unique chromatic signature all together in one single device. The structure consists of a resonant cavity, where the top mirror is replaced with a layer of plasmonic Ag nanoislands. The naturally random spatial distribution of clusters and nanoparticles formed by this deposition technique constitutes the manufacturer-resistant nanoscale morphological fingerprint of the proposed PUF. The presence of Ag nanoislands allows us to tailor the interplay between the photonic and plasmonic modes to achieve two additional security levels. The first one is constituted by the chromatic response and broad iridescence of our structures, while the second by their rich spectral response, accessible even through a common smartphone light-emitting diode. We demonstrate that the proposed architectures could also be used as an irreversible and quantitative temperature exposure label. The proposed PUFs are inexpensive, chip-to-wafer-size scalable, and can be deposited over a variety of substrates. They also hold a great promise as an encryption framework envisioning morpho-cryptography applications.

## Introduction

In a study called “Trends in Trade in Counterfeit and Pirated Goods”, the Organization for Economic Co-operation and Development (OECD) certified that the trade in counterfeit goods settles around 3.3% of global world trade, in 2019. Such a trend is rapidly increasing. The new emerging technological scenarios, like 5G, the internet of things, and distributed ledger technologies, require a new approach to secure intellectual properties as well as the authenticity of the traded goods. In this respect, a great promise is held by the so-called physical unclonable functions (PUFs).^1−3^ PUFs are individual physical signatures whose intrinsic unpredictability produces a unique and unclonable response to a specific challenge. In general, a PUF is a physical system whose internal structure manifests random disorder and uncontrollable manufacturing variations which make it unique and “unclonable” for users, counterfeiters, and even for the original PUF-manufacturer. PUFs can be interrogated following a challenge-response scheme. Depending on the number of independent challenge-response pairs it offers, a PUF can be categorized as “weak” or “strong”, according to a recently proposed taxonomy.^4^

The fulcrum over which a PUF leverages is constituted by the unavoidable randomness introduced in manufacturing processes, over which the operator or the designer has no control. In this respect, micro- and nanofabrication processes are the ideal reservoirs to look for user-independent randomness because, despite their reliability in terms of device operation functionalities, common lithographic techniques introduce a certain level of unpredictability.^5,6^ A PUF respecting such prerequisites is often regarded as a manufacturer-resistant PUF and holds the highest level of reliability.^3^ As a result, silicon-based metal-oxide-semiconductor (MOS) technology offered historically the first examples of PUFs.^7,8^ Field-effect transistors, even if equipped with profound structural innovations, still remain the platform of election for the embodiment of PUFs.^9−15^ It is however true that the always increasing necessity to encrypt and authenticate goods, in which transistors, ring-oscillators, or electronic systems embedding CMOS-related PUFs do not find place, makes it paramount to look for innovative materials and strategies.^16^ In this respect, the interaction between light and matter constitutes a fertile ground over which new approaches to PUFs are being developed, sometimes called “optical PUFs”.^17^ Optically weak PUFs have been proposed employing different materials and relevant examples, including nanostructures like nanoparticles,^18^ random silver nanowires,^19^ or organic nanolaser arrays.^20^ Countless chemical processes have been harnessed as well to generate tags of different natures as optical PUFs.^21,22^

Fluorescence has been widely used as an optical anticounterfeit function.^23−26^ Photoluminescence occurring in the perovskite^27^ and inkjet-printed quantum dots^28^ has been recently used as a PUF. Biological systems consisting of colonized populations of T-cells have also been demonstrated as high-security level biological unclonable functions.^29^

Colors represent a powerful tool for labeling, sometimes univocally characterizing the identity of the goods they are associated with. Red-Ferrari, for example, is the most recognizable feature of the brand, representing a strong authentication feature *de facto*. Colors, iridescence, and holography have been recognized to be so effectively strong in protecting and authenticating that practically the totality of banknotes adopts a color-related authentication label as one of the security gate-keepers.^30^

The world of nanotechnologies offers, in this respect, unique possibilities. In particular, fields of nanotechnology in which peculiar chromatic effects stem from unusual properties of nanometric resonators are of interest for this purpose.^31−44^ They, indeed, intrinsically offer the possibility of a double signature: (i) the chromatic one, represented by the specific colorful response of the system, and (ii) its spectral response.^45,46^ While the former is very easy to challenge by a quick visual investigation, the latter requires slightly more sophisticated approaches which often rely on the spectroscopic analysis of the label. The so-called plasmonic materials belong to this family. The capability of plasmonic nanoparticles (PNPs) to be used as anticounterfeit labels has, indeed, been recently investigated. Localized coherent oscillations of electrons occurring in PNPs endow them with a resonant and colorful optical response that has been widely used all across human history, more or less consciously.^47,48^ The chromatic response of PNPs, together with their size and random positioning over a substrate, constitutes a powerful triple approach to produce a robust PUF.^46^ However, to optimize the performance as PUFs and introduce a significant aleatory in their disposition over a substrate, very dispersed PNP solutions have to be used, so that their interdistance and clustering remain random, as recently shown by Torun et al.^49^ Under these conditions, the macroscopic chromatic response results in faint colors that necessitate a dark-field microscopy investigation to be fully appreciated, ruling out the possibility to carry out a simple and quick naked-eye challenge.^46^

**Figure 1:**
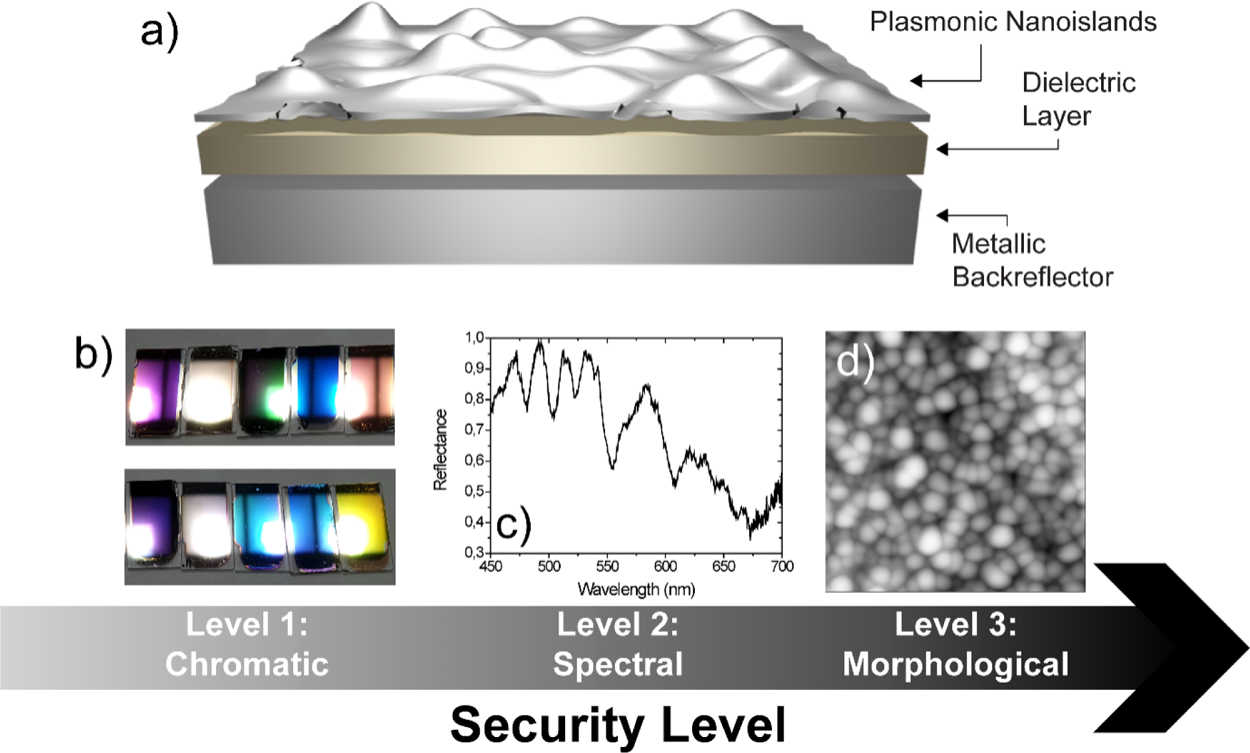
(a) Sketch of the plasmonic PUF architecture. (b) Chromatic and lightness effect being “Level 1” chromatic signature. (c) Typical spectral response of a sample resembling the architecture shown in (a). This constitutes the “Level 2” spectral signature. (d) Ag nanoisland morphology revealed via AFM analysis, constituting “Level 3” nanoscale morphological signature.

In this paper, we build on the concept of exploiting the plasmonic properties of nanostructured metallic surfaces in a hybrid plasmonic system engineered to be a three-level strong PUF. The proposed architecture is sketched in Figure 1a. The plasmonic PUF is structured to provide three different authentication levels: (i) chromatic (Figure 1b), (ii) spectral (Figure 1c), and (iii) morphological (Figure 1d). This represents also one of the main advancements offered by our approach with respect to the state-of-the-art devices because small-scale devices have usually been thought to be unsuitable as strong PUFs due to the intrinsic difficulty in introducing multilevel challenge-response pairs in a nanometric device.

Here, the paradigm shift resides in scaling the challenge-response pairs from macroscopic to nanometric effects rather than the device itself. The technological core of the idea proposed hereafter lies, indeed, in Ag nanoislands obtained by depositing Ag layers below their percolation threshold, via a DC magnetron sputtering technique.^50−55^ Such a procedure allows one to obtain Ag nanoclusters whose density and size depend on the sputtering deposition parameters but whose spatial disposition and clustering results are random. There is, indeed, no possibility for the operator to gain control over this feature, which, therefore, constitutes the ideal morphological fingerprint. Moreover, Ag nanoislands unify a marked chromatic response with a peculiar plasmonic behavior. Here, we demonstrate that when used as the top layer in a multilayered configuration involving also one or more metal/insulator/metal (MIM) resonators, the interplay between photonic cavity resonances and plasmonic modes gives rise to a glaring and distinct chromatic response. We characterized it in both the CIE 1931 and the CIELAB color space, demonstrating that color tints with a broad variety of lightness–hue–saturation values can be obtained. Such a property, combined with a marked iridescence, gives rise to a chromatic response that is unique and cannot be reproduced by commercial paints, representing level 1 of authentication of the proposed PUF. This first level is also the one thought to be readily accessible to the customer. The exceptional chromatic properties manifested by our samples stem from their spectrally rich optical response (Figure 1c), which we characterized both by sophisticated ellipsometric measurements and by using a light-emitting diode (LED) flash lamp of a smartphone in a simple spectroscopic setup. This latter investigation demonstrates the capability to engineer portable spectroscopic systems with which to equip a smartphone and provide the customer easy access also to the spectral fingerprint. In the end, the strongest and most innovative authentication level is given by what we called the morphological fingerprint, which is constituted by the spatial disposition of Ag nanoislands (Figure 1d). The morphology of a predetermined precise area of the surface of a sample on top of which Ag nanoislands have been deposited via sub-percolation threshold sputtering deposition has been investigated through atomic force microscopy (AFM) measurements. The outcome image has been registered as a fingerprint to be compared via a neuromorphic imaging recognition algorithm with those of different areas. We found out that the number of tags recognized by the algorithm by comparing two images of the same area, acquired with two different AFM measurements (positive recognition), is more than 2 orders of magnitude larger than the number of tags recognized by comparing AFM measurements belonging to two different areas of the patterned surface (negative recognition). The distance between the two expectation values of the related distributions is one of the larger reported so far, confirming the exceptional robustness of the proposed technique to false positive and false negative recognitions. We also simulate the critical experimental conditions in which the AFM operator could, by mistake, non-concentrically rotate the sample before performing the AFM measurement. Even in this case, our technique ensures an expectation value of recognized tags in the positive recognition process more than 1 order of magnitude larger than the negative recognition case. In the end, we demonstrate that our plasmonic PUF could work as a thermal exposure label. We show, indeed, that, when the sample is exposed to temperatures higher than a certain threshold, its color and optical response change dramatically and irreversibly in a deterministic and quantitatively measurable way.

The new concept reported in this article could potentially impact the PUF blockchain, introducing a new way of securing a broad variety of goods because this architecture could potentially be evaporated over every kind of metallic substrate acting as a back-reflector. The deposition technique is readily scalable from nano-to-wafer size, opening to the labeling of every kind of substrate from chip-embedded to commercial electronics device size. The possibility to produce plasmonic nanoislands is not a prerogative of Ag. All kinds of metals could be used, such as Al and Cu, for example, to achieve inexpensive plasmonic labeling. In the end, the exceptional randomness of the proposed surfaces holds great promise in the field of cryptography, as recently shown by Leem et al.,^56^ and could be potentially used as a morphological encrypting matrix to carry out morpho-cryptography.

## Results and Discussion

### Level 1: Chromatic Signature

**Figure 2:**
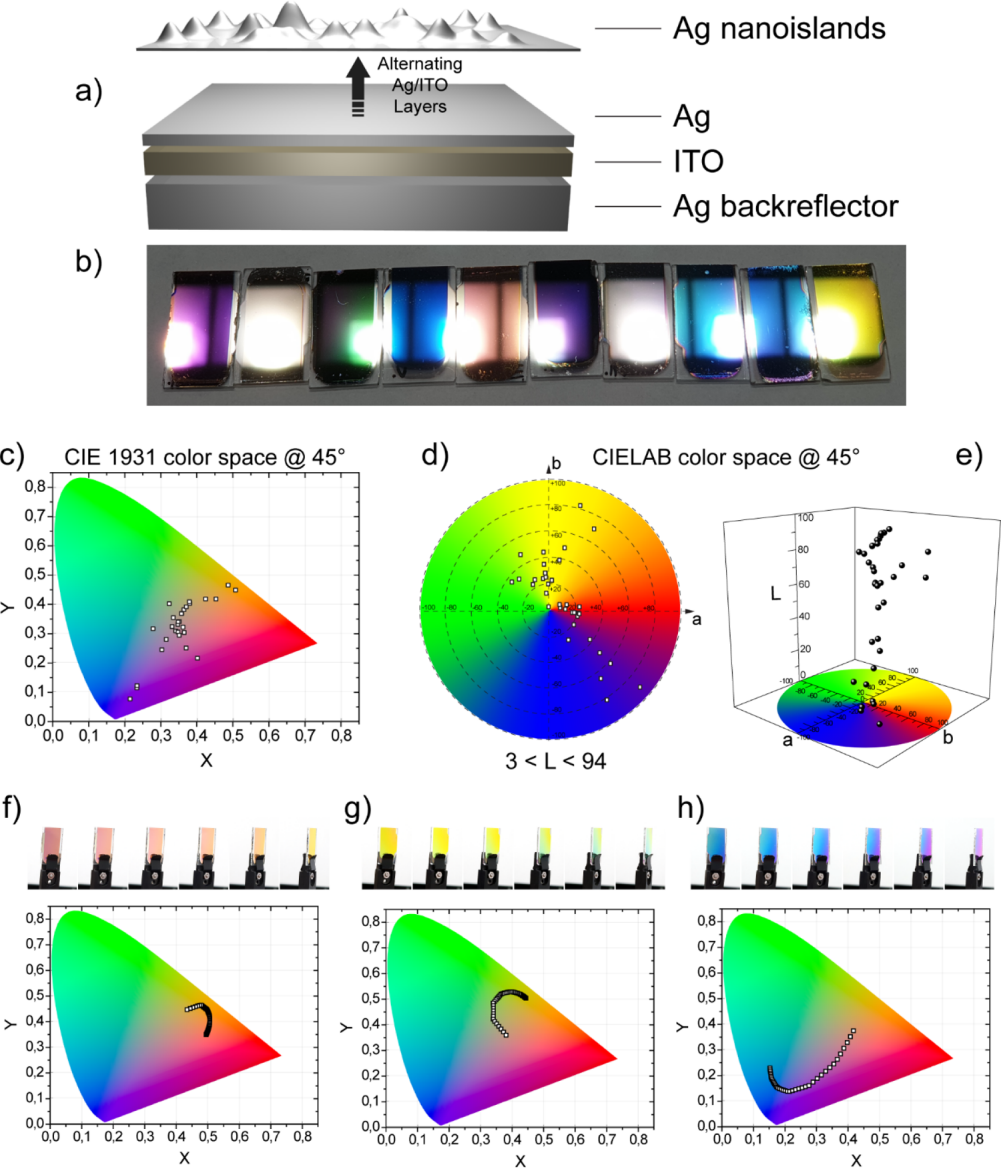
(a) Sketch of the MIN-multilayered configuration. (b) Picture showing several MIN structures selected to highlight the broad chromatic palette achievable with this architecture. The picture highlights also the metalized character of the obtained colors. The features appreciable in (b) can be quantified by means of (c) CIE 1931, (d) 2D, and (e) 3D CIELAB color space analyses. (c–e) transfer in the CIE notation. The measurements carried out at 45° for different MIN structures, changing either the thickness of the ITO layer or the number of cavities, allowing to realize the vast hue–saturation–lightness combinations. (f–h) Angle-dependent chromaticity (iridescence) of three significant MIN structures whose values are centered in red (f), yellow (g), and blue (h) color gamma, together with the CIE 1931 color space analysis of their angle-dependent chromaticity (iridescence).

Ag nanoislands manifest a marked size-dependent chromatic response. The size of Ag nanoislands can be easily controlled by changing the sputtering deposition time, as shown in Section S1 of the Supporting Information. The chromatic response of Ag nanoislands stems from their plasmonic properties. As such, the larger their size, the more the resonance shifts toward the red band of the optical spectrum. Despite the broad tunability of the plasmonic resonance of Ag nanoislands, the hue and saturation levels of the obtained colors are quite limited. Such a feature, together with a very low lightness, contributes to provide a “matte” color nuance. A far more variegated color gamma, together with a broad angle-dependence (iridescence), can be obtained by composite resonant structures made of one or many MIM multilayered cavities in which the top metallic layer is replaced with Ag nanoislands (Figure 2a). Metal/dielectric/nanoisland (MIN)-multilayered structures allow one to tailor the interplay between proper Fabry–Perot modes, hosted by MIM cavities, and plasmonic modes sustained by Ag nanoislands. Such an interaction can be controlled by acting on both the Fabry–Perot and the plasmonic sides. The former task can be accomplished by playing with: (i) the number of resonators (MIM cavities by which the entire structure is composed),^57^ (ii) thickness, and (iii) the refractive index of the employed dielectric layers.^58−60^ The latter aim could be pursued by changing the sputtering deposition time of the nanoislands. A decrease of the thickness and/or refractive index of the dielectric layer induces a blue shift of the resonance of the single MIM resonator.^58,60−65^ A variation of the number of multilayers by which the MIN multilayered is composed, enriches the chromatic response, fostering the insurgence of novel resonant modes through cavity mode hybridization.^57,59,66,67^

In the end, longer sputtering deposition times induce a redshift of the plasmonic resonance of the Ag nanoislands (see Section S1 of Supporting Information). The interplay between Fabry–Perot and plasmonic resonances endows the multilayered MIN structures with a variegated chromatic response, which is perfectly captured in both the CIE 1931 (Figure 2c) and CIELAB color space (Figure 2d,e), drawn from measures carried out at 45° by changing either the thickness of the ITO layers or the number of cavities.

The CIELAB diagram (Figure 2d,e) is particularly effective in capturing the chromatic versatility of the proposed nanostructures, demonstrating the possibility to cover practically the entire saturation (radius of the circle) and luminescence (*Z*-axis of Figure 2e) range. It is, on the contrary, more complicated to obtain a green tint. This is mainly due to the physics governing the formation of resonances in such samples. To obtain a green tint, indeed, full absorption in both the blue and red ranges has to be provided together with sharp reflectance in the green range. However, even though obtaining a sharp reflectance peak in the green range is quite easy, it is very difficult to suppress the red and the blue components simultaneously. As a result, it is very easy to obtain hues in the blue, violet, and yellow ranges, while obtaining pure green tints is rather difficult. The sophisticated chromatic scenario just described constitutes a distinct yet very hard to counterfeit signature of a potentially labeled object. The proposed structures, however, intrinsically possess a deeper security level which is constituted by their iridescence. Iridescence can be understood as the dependence of the chromaticity of an object on the observation angle. In our structures, this feature immediately translates into a modification of the reflectivity as a function of the angle. Figure 2f–h shows how the perceived color of the three representative structural changes with the observation angle. To showcase such a feature, we selected three structures. The one shown in Figure 2f manifests the color hue in the red range, while the ones in Figure 2g,h show in the yellow and blue ranges, respectively. All of them undergo a marked color hue change while being rotated (see the stop motion sequence, made of six images for each sample, on the top of the corresponding color space map). To quantify such an effect, we drafted the CIE 1931 color space characterization for each sample. In such an analysis, each point corresponds to the CIE value of the color of the sample obtained at a particular angle, from 0 to 90° angle.

The broadly customizable chromatic response, together with their iridescence, constitutes the first level of the complete plasmonic PUF. Reproducing, with a common paint, the particular lightness–hue–saturation characteristics of the colors obtained via our plasmonic architectures is, indeed, extremely difficult and it would be impossible to obtain the same iridescence.

### Level 2: Spectral Signature

**Figure 3:**
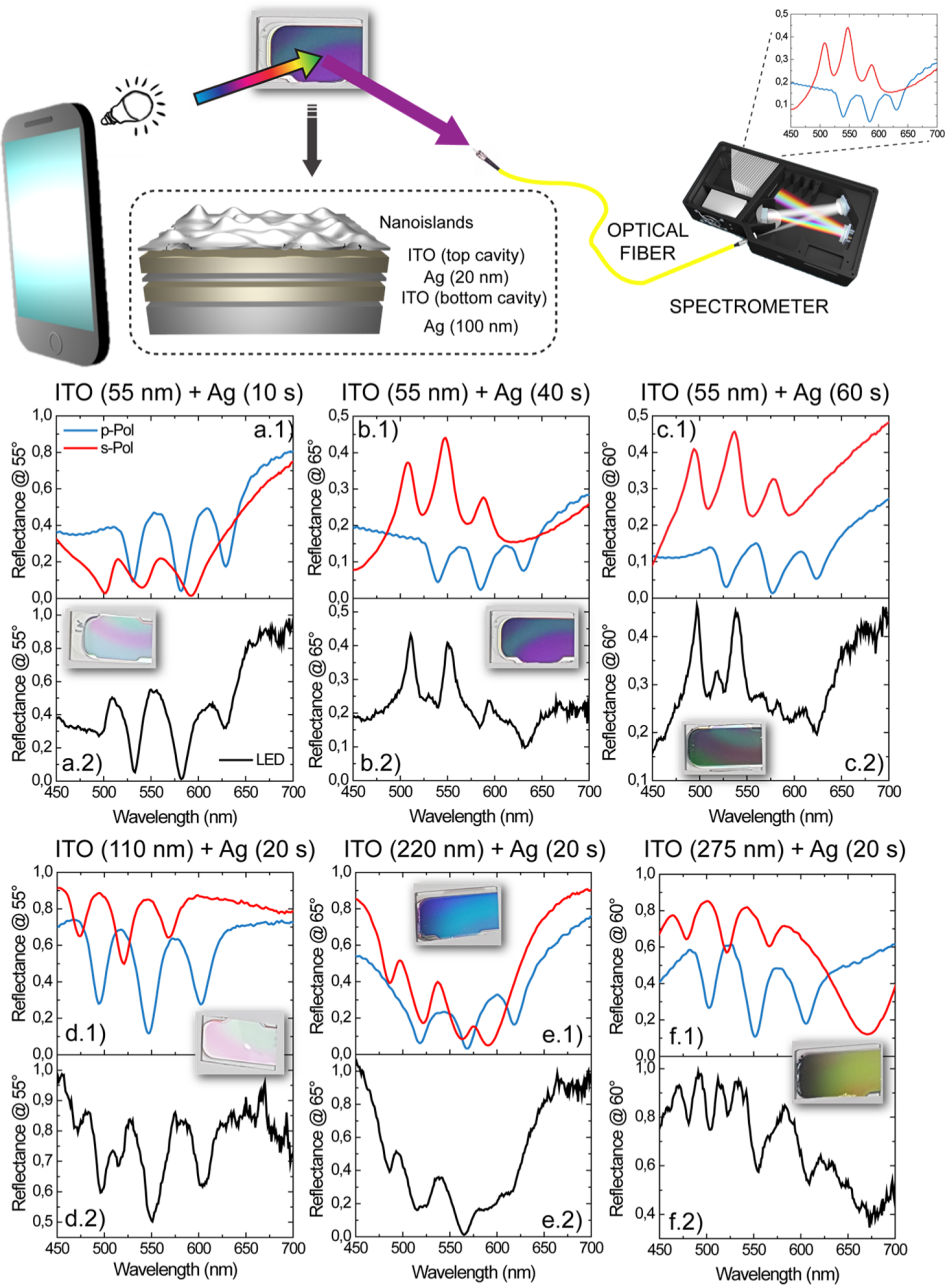
Sketch of the level 2—spectral signature challenging via simple illumination with a common smartphone LED flash lamp compared with ellipsometric measurements. Light emitted by the smartphone LED is directed onto the double-cavity MIN sample (a sketch of which is provided beneath the photo of the real sample) and reflectance is collected by a common spectrometer. (a1–f1) Ellipsometric analysis of the reflectance spectra of six different and mostly representative MIN structures, compared with (a2–f2) spectral analysis of the same samples measured by illuminating with a smartphone LED flash lamp.

The unique chromatic response of the multilayered MIN systems stems from their scattering spectral response. This means that their far-field scattering parameters (transmittance and reflectance) assume a rich and unique spectral shape. Each color corresponds to a specific spectral signature, constituting the second level of the complete plasmonic PUF. The level 2 signature could be challenged by simply illuminating it with a known light source, which, for this purpose, could also simply be constituted by the flash lamp of a commercial smartphone, as shown in the sketch of Figure 3a. The response to the challenge is given by its spectral signature. In our experiments, we investigated the spectral response of the produced multilayered MIN architecture by illuminating them using the flash lamp of a smartphone and compared the obtained results with those acquired by a rigorous analysis carried out *via* spectroscopic ellipsometry. To make our analysis independent from the emissivity of the LED flash lamp as well as on the environmental light conditions, we recorded it as a baseline to carry out a proper spectroscopic analysis. Examples of the most representative architectures are shown in Figure 3a–f. In particular, the reflectance spectra collected by an ellipsometric characterization of the six representative samples are reported in the upper box of each panel of Figure 3a1–f1, compared with the spectra acquired by using a smartphone LED flash as a light source, as illustrated in the bottom figure of the same panels (black curves in Figure 3a2–f2). The resonant behavior of the MIN multilayers holds a remarkable dependence on the angle of the impinging light, as described in the previous section. In addition, both the plasmonic and photonic nature of the compound multilayers introduces a marked dependence on the polarization of the impinging light. To simplify the handling of this concept, in Figure 3a1–f1, we show the reflectance spectra of the considered structures at one precise angle (being confident that the reader would rely on the iridescence of these structures provided in the previous section to figure out that the resonances of each sample blueshift, while increasing the impinging angle). The systems shown in Figure 3 consist of metal/dielectric-coupled double cavities, in which Ag nanoislands are used as the top layer, ITO as a dielectric spacer, and Ag as both the bottom and intermediate layers, as sketched in Figure 3. Such a configuration offers the higher degree of design flexibility because it allows one to play separately with both the thickness of the cavities through the ITO layers (photonic effect) and with the surface density of Ag nanoislands through the sputtering deposition time (plasmonic effect). On closer inspection, the double-cavity system would provide many additional degrees of freedom such as the coupling between the two cavities that can be controlled via both the thickness of the central metal layer and/or that of one of the ITO layers, as shown in previous works.^57,68^ We, however, restricted our analysis to the first two cases (photonic and plasmonic effect), which are the most salient for the proposed application.

In particular, from Figure 3a–c, we analyze the plasmonic effect. Here, the thickness of the ITO dielectric layers composing of the two cavities is fixed at 55 nm, while the surface density of the Ag nanoislands on top is varied accordingly with the sputtering deposition time. From Figure 3d–f, we analyze the photonic effect. Here, on the contrary, the surface density of Ag nanoislands is fixed (sputtering deposition time equal to 20 s) but the thickness of both the ITO layers changed from 110 to 220 nm and, in the end, to 275 nm. Both the effects allow obtaining very different spectral responses which access countless spectral signatures. It is however true that acting on the thickness of cavities (photonic effect) is the mechanism that produces the most significant modification in the spectral signature because it allows one to modify the number of resonances occurring in the visible range the way it happens for a classic Fabry–Perot resonator.

A quick comparison between the ellipsometric analyses and those carried out by illuminating with a LED flash lamp reveals that, as expected, in the case of the LED illumination, the spectral response is the result of the convolution of the p- and s-polarization reflectance (which have been rigorously discerned by the ellipsometric analysis). This endows the samples with a unique and rich spectral response, in which the reflectance dips and peaks acquired at a precise angle are positioned at well-determined wavelengths. Replicating such a spectral signature with an opaque, commercially available paint would be impossible because their opacity and very scattering surface would compromise the measure itself. In the case of metallic paints, the reflectance of which could, on the contrary, be experimentally measured, it would however be impossible to reproduce the typical resonant response of the MIN multilayers because metallic paints do not leverage on a resonant mechanism to produce their tints.

### Level 3: Morphological Signature

**Figure 4:**
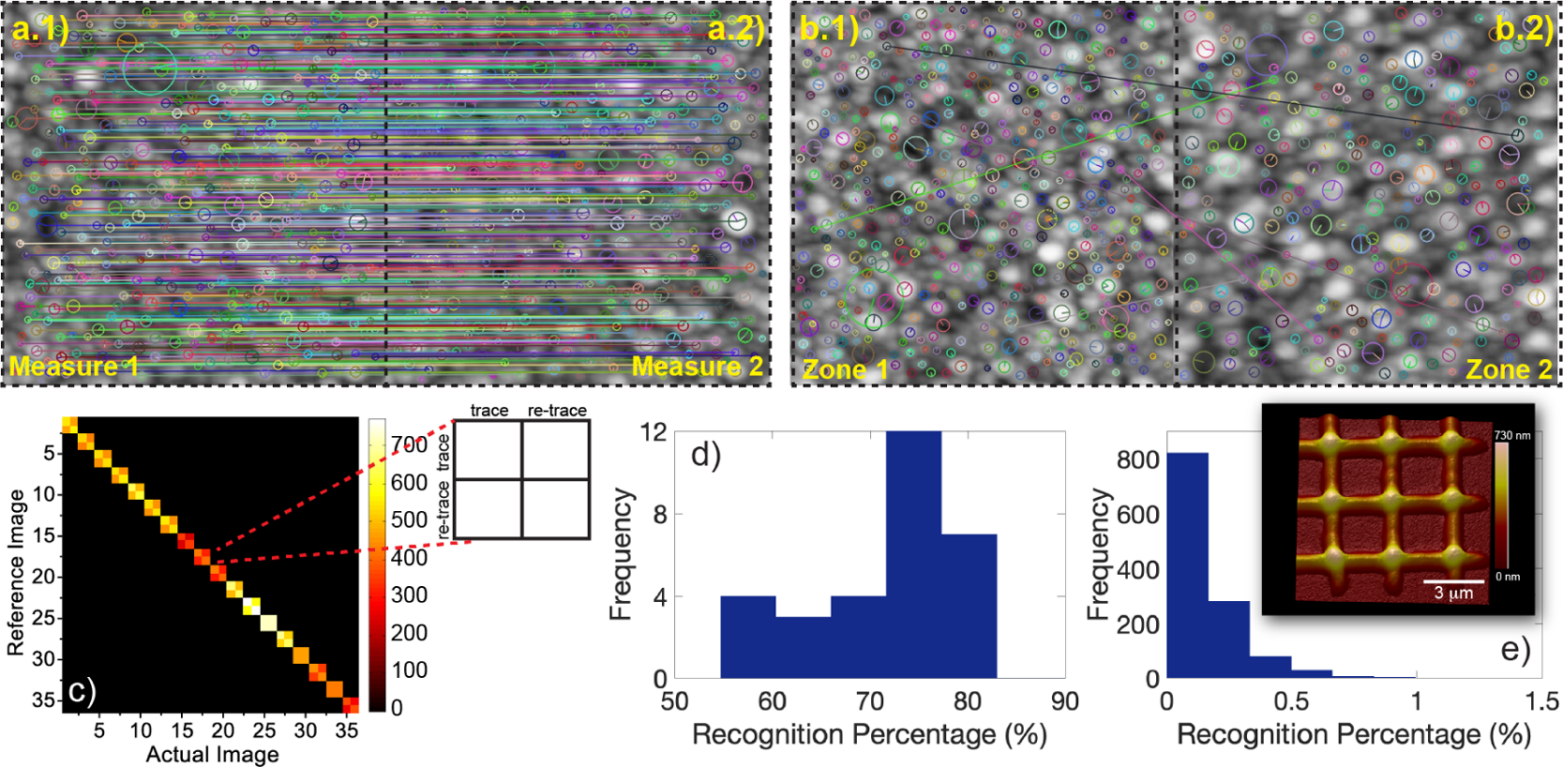
Comparison between (a.1) trace and (a.2) re-trace AFM measurements of the same area. (b.1,b.2) Comparison between AFM measurements of two different areas. (c) Score-Match matrix representing all the possible comparisons between all the analyzed areas. In the zoom, a sketch of the on-diagonal element of the Score-Match matrix constituted by a 2 × 2 matrix accounting for the trace/trace, trace/re-trace, re-trace/trace, and re-trace/re-trace comparisons of the same area. (d) Histogram of the distribution of the comparison between the AFM images belonging to the same area together with (e) comparisons of AFM measurements of different areas. In the inset, AFM measurement of a portion of the 3 × 3 μm grid into which the surface of a Ag nanoisland layer has been divided is shown.

The third and strongest level of the complete plasmonic PUF is represented by the nanoscale morphology of the Ag nanoisland layer deposited on top of each MIN multilayer. As said before, when an Ag layer with a sub-percolation threshold size is deposited via DC magnetron sputtering, it does not assume the features of a smooth film but, rather, its morphology turns into a rough assembly of nanoislands, manifesting peculiar collective resonant plasmonic properties. Both the density and the average size of the nanoislands depend on the deposition time, and the same is valid for their chromatic and plasmonic responses.^69^ In other terms, even though the number of nanoislands per μm^2^ and, therefore, the chromatic and plasmonic responses are determined by the sputtering parameters and remain repeatable, the morphological disposition of Ag nanoislands largely varies across the surface of the sample at a nanoscale level. What makes this signature unclonable is the fact that the operator has no control over the nanoscale morphology of a precise area of the sample, whose characteristics are not deterministically reproducible. A specific area of the sample, characterized by a peculiar nanoscale morphology, can, therefore, be taken as a manufacturer-resistant morphological barcode. The nanoscale morphology of a sample surface can be analyzed with great accuracy by means of AFM investigations. Such a technique is a standard investigation tool and, different from electron- or ion-beam-based imaging techniques, does not require any particularly stringent experimental constraint, such as high vacuum chambers, metallization, or high-voltage exposure. Here, AFM analysis is used to challenge the PUF, while its morphology constitutes the response. To prove the validity of Ag nanoislands as a morphological PUF, we sorted the top surface of a MIN multilayer as a grid, by lithographing a matrix of 3 × 3 μm squares with *i* rows and *j* columns, so that each investigation area is well defined and recognizable (inset of Figure 4e). The specific arrangement of the Ag nanoislands in each square determines its morphological barcode. Each square of the matrix has then been characterized via AFM measures both in trace and retrace modes. In the end, all the AFM measurements have been digitalized to allow the conversion of salient morphological features into tags to be recognized while comparing two different morphological images. In the end, all the morphologies of the squares have been compared with each other by means of open-access image recognition software (see the Methods/Experiment section). In particular, the AFM images are digitized and analyzed to evaluate relevant features for the comparison procedure (highlighted with colored circles in Figure 4a.1,a.2). The software then tries to recognize the same features between the two images of interest. Every time a match is confirmed, the software connects the two features with a line whose steepness carries information over the rotation of the image. A correlation matrix (sometimes called “Match-Score matrix”^17^) listing all the matching results in terms of recognized morphological features (tags) is then produced. The Match-Score matrix is one of the easiest and more widely used visual techniques to evaluate the validity of a PUF.^17^ An example of software comparison between two AFM measurements over the same area is provided in Figure 4a (a.1,a.2), while an example of the comparison between two AFM measurements carried out over two different areas is shown in Figure 4b (b.1,b.2).

The number of features recognized in the case of the comparison between two measures carried out over the same area (Figure 4a.1, AFM trace, Figure 4a.2 AFM re-trace) is very high as demonstrated by the large number of connecting lines in Figure 4a. On the contrary, a comparison between two AFM images belonging to two different areas carries very few recognitions, as stated by the paltry number of connecting lines occurring between the two images of Figure 4b.1,b.2. The Match-Score matrix associated with the recognition procedure carried out over all the measured areas is reported in Figure 4c. The matrix has to be read considering that equal *i*th and *j*th elements correspond to a trace AFM measurement of the same area, if *i* and *j* are odd numbers, and to a re-trace AFM measurement, if they are even. Therefore, the *i* + 1 to *j* comparison corresponds to a re-trace to the trace comparison of the same area and, finally, *i* to *j* + 1 comparison represents a trace to re-trace comparison of the same area. The elements on the diagonal of the Match-Score matrix are, therefore, 2 × 2 sub-matrices structured, as shown in the zoom of Figure 4c. Off-diagonal elements correspond to comparisons between two different areas. The color of each *i*–*j* pair corresponds to the number of recognized features. Noticeably, the features recognized on the diagonal of the Score-Match matrix are more than two orders of magnitude larger than the number of features recognized in an off-diagonal comparison.

**Figure 5:**
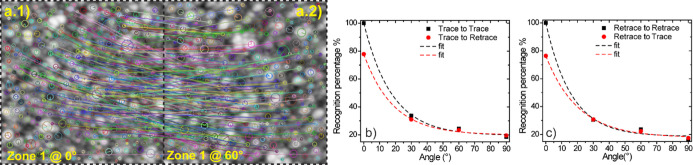
Example of a comparison carried out by the imaging recognition software between the AFM image of a precise area of a Ag nanoisland surface (a.1) and that of the same area tilted by 60° (a.2). Percentage of features recognized while rotating the sample in all the four possibilities: (b) trace-to-trace and trace-to-retrace and (c) retrace-to-trace and retrace-to-retrace.

Starting from the information acquired with the Match-Score matrix, we can define as a figure of merit the “tag recognition expectation value” (TREV), corresponding to the expectation value of the distribution of the number of tags recognized while comparing two different morphological areas. This parameter characterizes the capability of the nanoscale morphological fingerprint to work as a strong PUF. The distribution of the recognized tags for the cases in which a trace (retrace) square is compared with itself and/or with its retrace (trace) counterpart is shown in Figure 4d. The TREV of the distribution is about 76%, while the minimum of recognized tags in these cases is 55%. The number of recognized tags while comparing two different areas, no matter the trace/retrace acquisition, does not exceed 1%, with a TREV value of 0.11% (Figure 4e). The two distributions do not show any overlap, highlighting the solidity of such a technique. The robustness of the Ag nanoislands to false-positive recognitions becomes glaring when comparing the two TREVs. The one inherent to the comparison of two different AFM scans of the same area is 690 times larger than that inherent to a comparison between two different areas, leaving practically no margins for false positive recognitions. Envisioning a practical application as a PUF, the most critical event corresponds to the case in which the correct area has been accidentally non-coaxially rotated by the operator while performing the AFM measurement. This could potentially lead to a missed recognition due to an error of the operator (a false negative). To simulate this case, we carried out AFM investigations while rotating the same area from 0 to 90°. As for the previous case, four possibilities are salient: (i) a trace-to-trace comparison, in which the reference AFM analysis has been carried out as a trace measure (AFM tip moves left-to-right) and the compared image corresponds to another trace AFM measure, (ii) a trace-to-retrace comparison, where the compared image corresponds to a retrace AFM (AFM tip moves right-to-left), (iii) a retrace-to-retrace comparison, where both the reference and compared images correspond to retrace AFM measurements and, finally, (iv) a retrace-to-trace comparison in which a retrace AFM image is taken as a reference, compared to a trace AFM measure. Cases (i) and (iii) correspond to the homogeneous cases, in which higher recognition values are expected. Cases (ii) and (iv) represent the mixed cases, and for them, lower recognition values should be expected. The imaging recognition algorithm is however able to discriminate if the same image has been rotated with respect to the original one, being able to correlate an acceptable number of features also in this case. Figure 5a shows the case of a 60° rotation of the same area (Figure 5a.1 for θ = 0°, Figure 5a.2 for θ = 60°), together with the connection lines between tags that the algorithm recognizes as equal.

We found that the number of recognized tags, *R*(ϑ), decreases with the exponential law *R*(ϑ) = *A*_0_·e^–(ϑ/τ)^ + *p*_0_ with respect to the rotation angle. Here, *A*_0_ corresponds to the tag recognition percentage at ϑ = 0°, ϑ is the rotation angle, τ is the decay constant, measured in (degrees)^−1^, and *p*_0_ is the offset of the exponential law, corresponding to the tag recognition value toward which the exponential law tends to settle. The aforementioned cases (i) and (ii), in which the comparison is carried out using a trace AFM measure, are shown in Figure 5b, while cases (iii) and (iv), in which a retrace AFM measure constitutes the benchmark, are analyzed in Figure 5c. The starting points of the homogeneous cases correspond to comparing the same image and, of course, the tag recognition percentage is equal to 100%. Surprisingly, the exponential law describing the tag recognition percentage as a function of the rotation angle shows an offset around 20% (*p*_0_ ≈ 20). In the worst case of a 90° rotation, the tag recognition percentage does not go below 18%. In the following table (Table 1), the values of the tag recognition exponential decrease law are reported:

**Table 1:** Fitting Parameters (Confidence Interval ±10%) Inherent to the Exponential Law Describing the Decrease of Recognized Tags While Rotating the Sample under the AFM Apparatus

comparison type	*A*_0_	τ	*p*_0_
trace to trace	80.39	17.73	19.55
trace to retrace	57.87	18.45	19.95
retrace to trace	81.33	16.17	18.61
retrace to retrace	58.53	20.40	17.81

### Irreversible Thermal Switching

**Figure 6:**
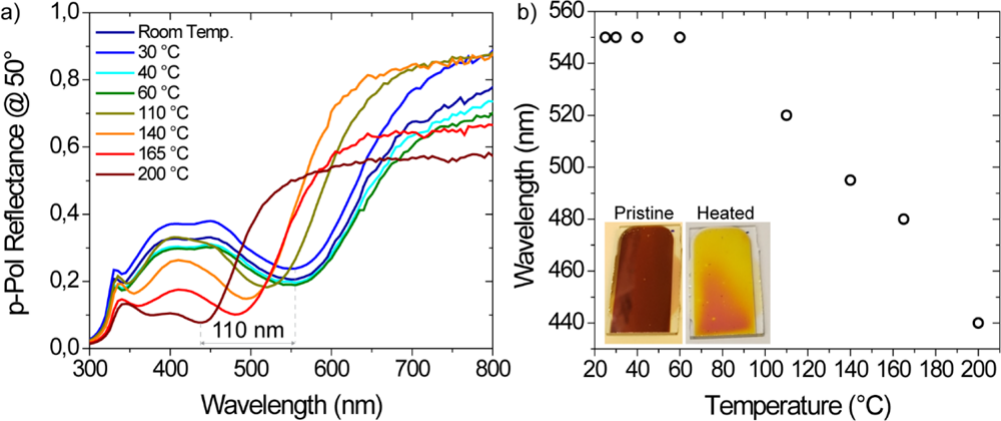
(a) Blueshifting of the p-polarization reflectance response of a MIN sample made, from bottom to top, of Ag (100 nm)/ITO (110 nm)/nanoislands (30 s) while increasing its temperature from room temperature to 200 °C. (b) Blueshift of the reflectance dip shown in (a) as a function of the temperature of the sample. In the inset, a photo of the pristine and heated MIN showing a marked color change.

A feature that is often demanded in security labels is the capability to track temperature exposure of the labeled products. The multilayered MIN architectures proposed in this work are the perfect candidates to accomplish such a task because Ag nanoislands undergo an irreversible oxidation process when exposed to high temperatures. As a result, the resonance of the MIN multilayer experiences a significant blueshift, as a function of the exposed temperature. In Figure 6a, we show the temperature-dependent p-polarization reflectance spectrum, acquired via temperature-varying ellipsometry (TVE) measurements inherent to a MIN structure made of Ag (100 nm)/ITO (110 nm)/nanoislands (30 s), from bottom to top. As summarized in Figure 6b, where the spectral position of the mode of the MIN structure is plotted against the temperature while heating the sample from room temperature to 200 °C, the main mode of the structure red-shifts to about 110 nm, with a knee at about 70 °C. The oxidation process of Ag nanoislands induces a remarkable refractive index change of the plasmonic elements (see Section S2 of Supporting Information), resulting in a dramatic and irreversible color change. Such a mechanism can be easily used as a visual irreversible marker of product exposure to high temperatures.

## Conclusions

In this work, we engineered a strong PFU based on a multilevel validity check process all of which included in the same platform. The architecture around which the PUF is designed consists of a composite multilayer in which a plasmonic element represented by Ag nanoislands interacts with a resonant one made of one or more MIM cavities. Such a hybrid plasmonic/photonic architecture manifests exceptional chromatic versatility, characterized by means of both CIE 1931 and CIELAB diagrams, that, united to a peculiar iridescence, constitutes the first recognition checkpoint, which we call “chromatic signature”. The specific chromatic response each structure is endowed with stems from a rich and detailed spectral response, resulting from the convolution of both the P- and S-polarized reflectance of the sample. The spectral response of the structure constitutes also the second recognition checkpoint, which we identify as the “spectral signature”. We demonstrate that, despite the sophisticated nature of such an investigation, it could be readily and easily carried out by illuminating the sample employing the LED flash lamp of a smartphone, envisioning the implementation of simple customer-level investigation tools ready to be integrated with commercial portable devices. In the end, as a third recognition checkpoint at the nanoscale, we exploit the random distribution of plasmonic nanometric units resulting from sub-percolation threshold sputtering deposition of Ag. Such a process gives rise to a rough surface distribution of Ag nanoislands, which can be investigated by means of AFM measurements. The AFM map can then be digitalized and used as a morphological nanoscale barcode acting as a recognition tag. The intrinsic impossibility of the operator to gain control over the morphological disposition of the Ag nanoislands ensures the unclonability of the morphological barcode, which can be classified as “manufacturer resistant”. We demonstrated by means of software image recognition techniques, the robustness of the morphology-based recognition process. In particular, we found that the TREV in the case of the comparison between two images inherent to AFM analyses of the same area (proper recognition) is about 76%, being 690 times larger than the TREV stemming from a comparison of AFM scanning of two different areas (counterfeit case). In the end, we demonstrate the robustness of the proposed PUF to errors potentially occurring while operating the AFM apparatus and, specifically, in the case in which the tag is non-coaxially rotated with respect to the predefined analysis position. Even in this case, a tag recognition percentage of 20% minimum is found, a value that is 180 times larger than the counterfeit TREV. The integrated and multipronged approach we propose here holds great promise in revolutionizing the PUF blockchains turning the randomness intrinsic in nanotechnology processes into the key to reach out to incomparable anticounterfeit labeling strength. The unique values of hue, saturation, and lightness obtainable with the proposed MIN multilayers foster their application in new-generation trademarks, which could intrinsically be endowed with robust anticounterfeit characteristics. Our plasmonic PUF can be easily deposited over any metallic back-reflector, opening to a cheap tagging of a plethora of devices and goods. The straightforward deposition technique by which our plasmonic PUFs are produced is scalable from the wafer-to-chip size, envisioning the integration of our tags in consumer-level electronics. In the end, the exceptional and irreproducible randomness of the nanoisland formation process can be potentially employed as a morphological cryptography platform, with a potential disruptive impact on novel-distributed ledger technologies, where high cybersecurity standards are paramount.

## Methods/Experiment

### Ag and ITO Sputtering Deposition

Multilayer Ag and ITO thin-film substrates were deposited *via* DC magnetron sputtering using the sputtering parameters reported in Table 2.

**Table 2:** Sputtering Parameters

material	power (W)	rate (nm/s)	pre-sputtering chamber pressure (mbar)	sputtering chamber pressure (mbar)
Ag	20	0.25	3 × 10^–5^	4.6 × 10^–2^
ITO	40	0.16	3 × 10^–5^	4.6 × 10^–2^

### Ag Nanoisland Sputtering Deposition

Ag nanoislands have been obtained *via* DC magnetron sputtering deposition of Ag layers with a sub-percolation threshold size. The obtained layers (characterized in Section S1 of Supporting Information) manifest a marked plasmonic response. The sputtering deposition parameters are:

1. Pre-deposition chamber pressure: 3 × 10^–5^mBar.
2. Deposition chamber pressure: 4.6 × 10^–2^mBar.
3. Deposition power: 12 W;
4. Deposition time: 5–60 s.

#### CIE 1931 and CIELAB Color space Analysis

CIE 1931 and CIELAB color space analyses have been carried out *via* a customized MatLAB code. We begin by measuring both p- and s-polarized measurements and considering the convolution of both spectra. The electromagnetic signal reaching the human eye after being scattered by the sample is, indeed, the result of the convolution of both the polarizations. In particular, after having introduced the tri-stimulus value functions, we determine both the XYZ and xyz coordinates of the CIE 1931 color space. We then plot the related diagram as shown in the main article. Then, once the CIE 1931 coordinates have been determined, we translate them into CIELAB parameters to determine, for each sample and at the desired angles, the values of hue, saturation, and lightness.

### Ellipsometrical and Smartphone LED Flashlamp Characterization of the Spectral Response of MIN Structures

p- and s-polarization reflectance measurements have been carried out over an ellipsometric setup (M2000 from Woollam), using a Xe lamp as a source. We then carried out a reflectance spectroscopic investigation using the LED flash lamp of a smartphone as a light source and collected the unpolarized reflectance spectrum at many angles (in the main paper we show only the most significant of them) using an Ocean Optics Flame spectrometer.

### Temperature Varying Ellipsometry

Temperature varying ellipsometry measurements have been carried out by equipping a VVase ellipsometer from Woollam with a custom hot stage produced by CaLCTec (Calabria Liquid Crystal Technology). We performed scattering and spectroscopic ellipsometry at each temperature specified in the main article of (i) bare Ag nanoislands, (ii) bare ITO layer, and (iii) the entire MIN structure. This allowed us to quantify the refractive index variation of both ITO and Ag nanoislands and evaluate their contributions in the spectral shift, as shown in Figure 6a. The pristine and heated refractive indices of both ITO and Ag nanoislands have been reported in Section S2 of the Supporting Information.

### Imaging Recognition Algorithm

Image recognition and feature matching have been carried out by means of a software application based on the Scale Invariant Feature Transform (SIFT) algorithm. SIFT is a computer vision algorithm for pattern recognition in 2D-image invariants to rotation, scale zooming, and brightness changing.^70^ The main steps are as follows: (i) constructing a scale-space, to make sure that features are scale independent; (ii) keypoint localization, to identify the suitable features or keypoints; and (iii) orientation assignment, to guarantee keypoint rotation-invariant; and keypoint descriptor, to assign a unique fingerprint to each keypoint.^71^

The keypoint detection consists of identifying locations and scales that can be assigned many times to the same object from different points of view. Locations, invariant to scale change of the image, can be detected by searching for features with stable values for all possible scales. A continuous function of scale, the scale space, defined as a function, *L*(*x*,*y*,σ) and formulated as the convolution of a variable-scale Gaussian, *G*(*x*,*y*,σ), with an image, *I*(*x*,*y*), is exploited to detect stable keypoint locations in the scale space. Each keypoint is featured with a consistent orientation based on local image properties, enabling the generation of a keypoint descriptor related to this orientation and therefore invariant to image rotation. The pixel difference allows computing the gradient magnitude, *m*(*x*,*y*), and orientation, θ(*x*,*y*). The correspondence among feature points in the original image and feature points in the input image can be evaluated, identifying, for each feature point, the nearest neighbor in feature vectors of the input image. The nearest neighbor is the feature point with a lower Euclidean distance for the invariant descriptor vector. A software library implementing the SIFT algorithm written in C++, available on https://opencv.org/, was employed to design a Java tool for analyzing and comparing the acquired images, allowing us to obtain the results reported in this paper.

### AFM Measurements

AFM measurements have been carried out using a Multimode 8 equipped with a Nanoscope V controller (Bruker). Data were acquired in the tapping mode, using silicon cantilevers (model TAP150, Bruker). Surfaces were imaged in air in a scan size of 1 × 1 μm and at different scan angles.

## Supporting Information Available

Morphological and plasmonic characterization of Ag nanoislands, oxidation of Ag nanoislands, and refractive index change (PDF)
