# Supplementary material for: Hybrid Plasmonic/Photonic Nanoscale Strategy for Multilevel Anticounterfeit Labels
Source: ACS Appl Mater Interfaces. 2021 Oct 11;13(41):49172–83. doi: 10.1021/acsami.1c13701 (PMC8532117; doi:10.1021/acsami.1c13701)
Supplement: Supplementary file 1 — am1c13701_si_001.pdf [file am1c13701_si_001.pdf]

SUPPORTING INFORMATION

A Hybrid Plasmonic/Photonic Nanoscale Strategy for Multi-Level Anti-Counterfeit Labels

Vincenzo Caligiuri[*,1,2], Aniket Patra[1,3], Maria P. De Santo[1,2], Agostino Forestiero[4], Giuseppe Papuzzo[4], Dante M. Aceti[5], Giuseppe E. Lio[6], Riccardo Barberi[*,1,2] and Antonio De Luca[*,1,2].*

[1] Department of Physics, University of Calabria, via P. Bucci, 31c, 87036, Rende (CS), Italy

[2] CNR Nanotec UOS Rende, via P. Bucci, 31d, 87036, Rende (CS), Italy

[3] Istituto Italiano di Tecnologia, via Morego 30, 16163 Genova (GE), Italy

[4] CNR-ICAR, Institute for High Performance and Networking, via P. Bucci 8-9c, 87036 Rende (CS), Italy

[5] Institute of Electronics, Bulgarian Academy of Sciences, 72, Tsarigradsko Chaussee blvd., 1784 Sofia, Bulgaria

[6] CNR-INO and European Laboratory for Non Linear Spectroscopy (LENS), Via Nello Carrara, 1 - 50019, Sesto Fiorentino, Firenze (FI), Italy

Corresponding Authors

*vincenzo.caligiuri@unical.it (ORCID: 0000-0003-1035-4702), *antonio.deluca@unical.it (ORCID: 0000-0003-2428-9075), *riccardo.barberi@fis.unical.it (ORCID: 0000-0001-9713-1696).

Keywords: physical unclonable functions, Ag nano-islands, plasmonics, metal-insulator-metal structures, iridescence, structural colors, temperature exposure sensors

SECTION 1: Morphological and Plasmonic characterization of Ag nano-islands

As explained in the main manuscript, Ag nano-islands can be obtained by depositing, *via* DC Magnetron sputtering, layers whose thickness is below the percolation threshold of Ag. This prevents the formation of a smooth and uniform film, while fostering the formation of randomly organized clusters of Ag nano-particles we called nano-islands. Increasing the sputtering deposition time brings to denser packing of nano-islands, eventually forming a uniform Ag layer. **Figures S1a-f** show Scanning Electron Micrograph (SEM) analysis of Ag nano-islands layers obtained *via* DC magnetron sputtering, with deposition time of (a) 5 s, (b) 10 s, (c) 15 s, (d) 30 s, (e) 40 s and (f) 60 s. The interaction between neighboring particles, together with the specific plasmonic resonance manifested by different sized clusters, contribute to provide a cooperative macroscopic plasmonic response whose signature is constituted by the reflectance dips shown in **Figure S1h,** occurring at the wavelengths highlighted in **Figure S1i**. From such a macroscopic plasmonic effect, a peculiar chromatic response is generated. The plasmonic response is, however, quite weak, leading to a recognizable but limited color gamut, as evidenced in the CIE 1931 diagram of **Figure S1l**, with very low saturation and lightness levels, as revealed by the CIELAB analysis shown in **Figure S1m**.

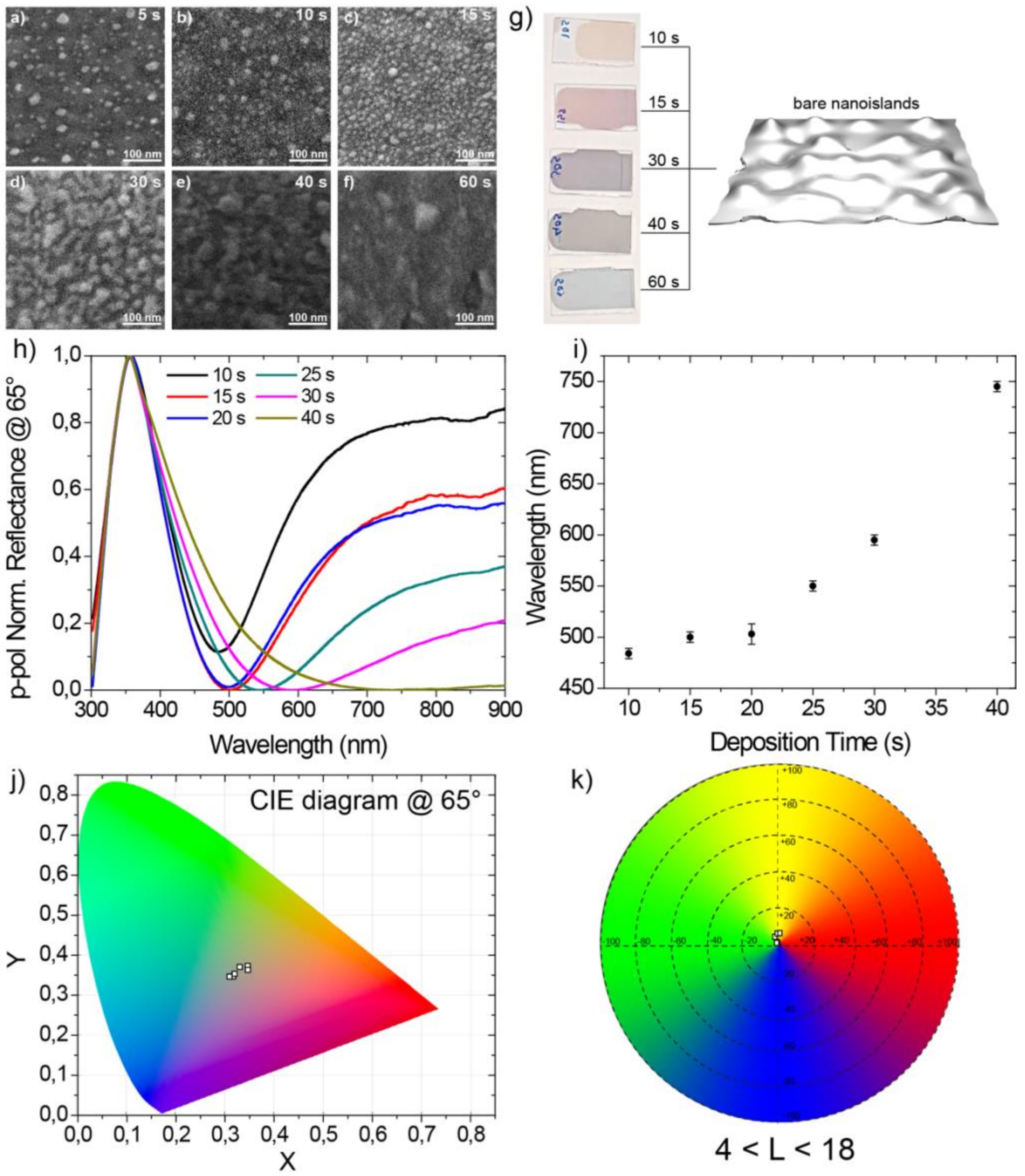

Figure S1. *(a-f) SEM analysis of six different Ag nano-islands layers at deposition times equal to 5 s, 10 s, 15 s, 30 s, 40 s and 60 s, respectively. g) Chromatic response of some of the samples analyzed before together with a 3D sketch of the Ag nano-islands layer. h) Normalized p-polarization reflectance for the Ag nano-islands at 10 s, 15 s, 20 s, 25 s, 30 s and 40 s measured at 65° angle of incidence together with (i) the spectral position of the reflectance dips associated*

to them. (j) CIE 1931 and (k) CIELAB hue-saturation diagram, together with an indication of the lightness range of all the Ag nano-islands layer analyzed before.

SECTION 2: Oxidation of Ag nano-islands and refractive index change

Ag nano-islands undergo an oxidation process if heated in room ambient. Such a phenomenon is responsible for the marked color change and blue-shift of the plasmonic resonance shown in the main manuscript, which can be used as a temperature exposure labeling. The oxidation of Ag nano-islands can be confirmed ellipsometrically. In **Figures S2a,b** a comparison between the measured refractive index of a Ag nano-islands layer, obtained with deposition time equal to 35 s before (blue curve) and after (red curve) the heating process at 200 °C, is shown. It can be immediately noted the occurring of a Lorentzian peak in the imaginary part of the heated sample around 450 nm. This corresponds to the bandgap of Ag_2O, formed as a consequence of the oxidation process of Ag nano-islands. In **Figures S2c,d** we report also the ellipsometric analysis and fit of the ellipsometrical angles Ψ and Δ, to highlight the good quality of the fitting procedure. To provide additional insight on the presence of a semiconductor bandgap, we report in **Figure S2e** the transmittance spectrum of the heated Ag nano-islands layer. A marked dip at 450 nm is visible. Moreover, from **Figure S2f,** which shows the s-polarization angular transmittance 2D map of the same sample, it is possible to see that the wavelength of the transmittance dip does not shift as a function of the angles. Being dispersion-less, we can confirm that it corresponds to the bandgap of a semiconductor, Ag_2O in the case in point.

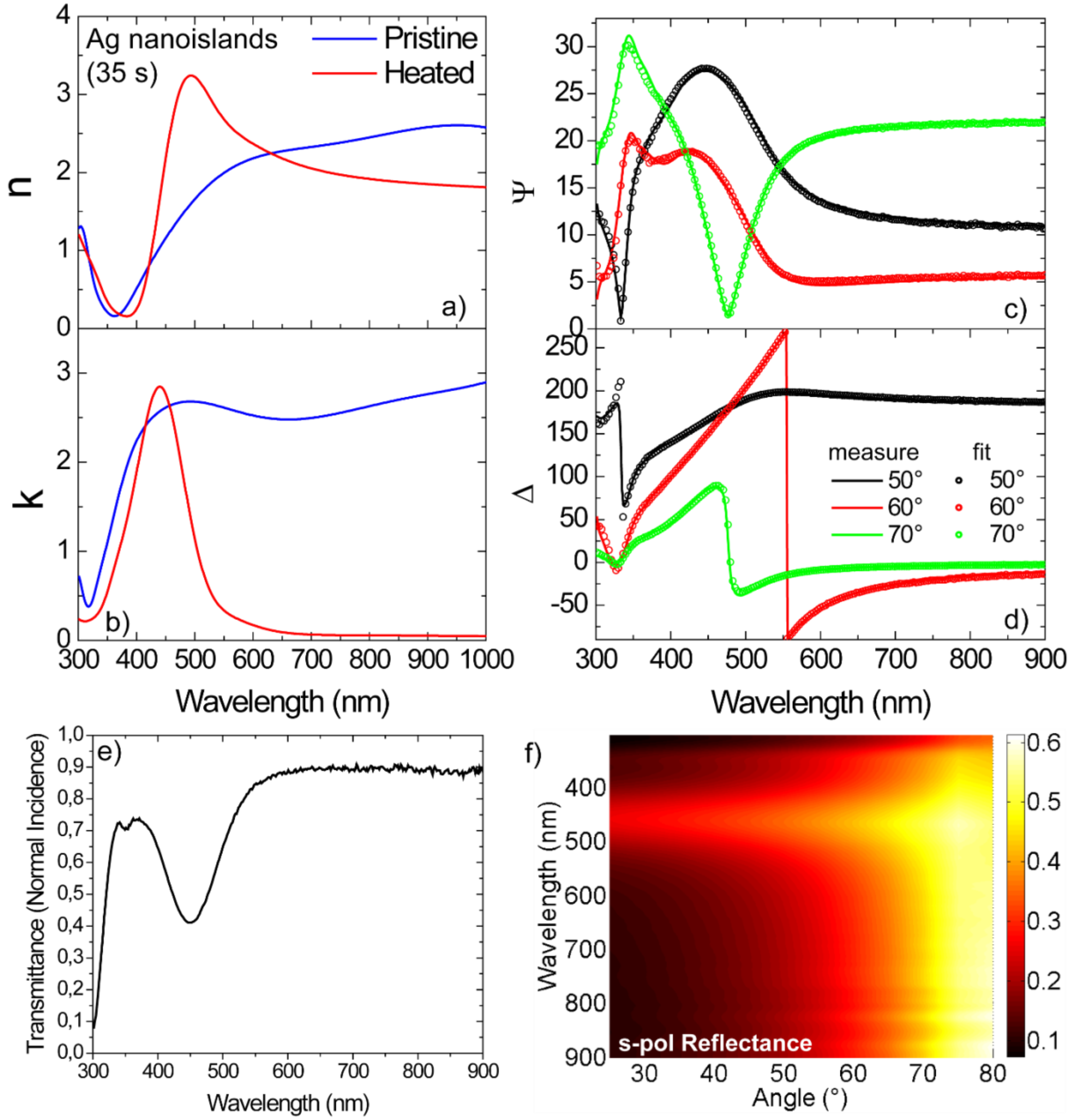

Figure S2. *(a-b) Real and imaginary refractive index of pristine (blue curve) and 200 °C heated (red curve) Ah nano-islands layer (deposition time 35 s), together with the measured and fitted ellipsometrical angles (c) Ψ and (d) Δ. (e) Transmittance of the Ag nano-islands layer (deposition time 35 s) together with (f) s-polarization angular reflectance map.*
